# Flexible Textile-Based Pressure Sensing System Applied in the Operating Room for Pressure Injury Monitoring of Cardiac Operation Patients

**DOI:** 10.3390/s20164619

**Published:** 2020-08-17

**Authors:** De-Fen Shih, Jyh-Liang Wang, Sou-Chih Chao, Yin-Fa Chen, Kuo-Sheng Liu, Yi-Shan Chiang, Chi Wang, Min-Yu Chang, Shu-Ling Yeh, Pao-Hsien Chu, Chao-Sung Lai, Der-Chi Shye, Lun-Hui Ho, Chia-Ming Yang

**Affiliations:** 1 eBio Technology Inc., Xinzhuang, New Taipei City 242, Taiwan; stephanie@ebio-health.com (D.-F.S.); joewang@mail.mcut.edu.tw (J.-L.W.); willson@ebio-health.com (S.-C.C.); DCS19680323@gmail.com (D.-C.S.); 2 Department of Electronic Engineering, Ming Chi University of Technology, New Taipei 243, Taiwan; 3 Institute of Electro-Optical Engineering, Chang Gung University, Taoyuan 333, Taiwan; chenbearfa@gmail.com (Y.-F.C.); cmyang@mail.cgu.edu.tw (C.-M.Y.); 4 Department of Cardiac Surgery, Chang Gung Memorial Hospital, Linkou 333, Taiwan; liuks@me.com; 5 Department of Nursing, Linkou Chang Gung Memorial Hospital, Linkou 333, Taiwan; robelae@cgmh.org.tw (Y.-S.C.); gigy@cgmh.org.tw (C.W.); yu@cgmh.org.tw (M.-Y.C.); q22122@cgmh.org.tw (S.-L.Y.); 6 Department of Nursing, Chang Gung University, Taoyuan 333, Taiwan; 7 Department of Nursing, Oriental Institute of Technology, New Taipei City 220, Taiwan; 8 Department of Nursing, Chang Gung University of Science and Technology, Taoyuan 333, Taiwan; 9 Department of Cardiology, Chang Gung Memorial Hospital, School of Medicine, Chang Gung University, 199 Tung Hwa North Road, Taipei 105, Taiwan; pchu@cgmh.org.tw; 10 Department of Electronic Engineering, Chang-Gung University, Taoyuan 333, Taiwan; cslai@mail.cgu.edu.tw; 11 Biosensor Group, Biomedical Engineering Research Center, Chang Gung University, Taoyuan 333, Taiwan; 12 Department of Nephrology, Chang Gung Memorial Hospital, Linkou 333, Taiwan; 13 Department of Materials Engineering, Ming-Chi University of Technology, New Taipei City 243, Taiwan; 14 Department of General Surgery, Chang Gung Memorial Hospital, Linkou 333, Taiwan

**Keywords:** cardiac operation, flexible pressure sensor, interfacial pressure, Kalman filter, pressure injury

## Abstract

Pressure injury is the most important issue facing paralysis patients and the elderly, especially in long-term care or nursing. A new interfacial pressure sensing system combined with a flexible textile-based pressure sensor array and a real-time readout system improved by the Kalman filter is proposed to monitor interfacial pressure progress in the cardiac operation. With the design of the Kalman filter and parameter optimization, noise immunity can be improved by approximately 72%. Additionally, cardiac operation patients were selected to test this developed system for the direct correlation between pressure injury and interfacial pressure for the first time. The pressure progress of the operation time was recorded and presented with the visible data by time- and 2-dimension-dependent characteristics. In the data for 47 cardiac operation patients, an extreme body mass index (BMI) and significantly increased pressure after 2 h are the top 2 factors associated with the occurrence of pressure injury. This methodology can be used to prevent high interfacial pressure in high-risk patients before and during operation. It can be suggested that this system, integrated with air mattresses, can improve the quality of care and reduce the burden of the workforce and medical cost, especially for pressure injury.

## 1. Introduction

Pressure injury is a high-risk issue for long-staying patients, the elderly, and disabled people with reduced daily activities if proper care is not available. The patients impacted by pressure injuries in the United States numbered approximately 2.5 million, following more than 60,000 deaths [1]. Due to healed wounds possibly leading to poor tissue properties, more than 65% of pressure injuries recur. The average cost for a patient with pressure injuries was more than $29,000 in 1998 [2] and increased to more than $44,000 in 2013 [3]. The occurrence of a pressure injury causes a more than 3.5-fold increase in the patient’s hospital length of stay. In the worst case, it may contribute to premature mortality. Some extra costs, including prostheses, loss of income and job, rehabilitation, disability payment, and potential litigation, should also be considered in the total impact to society. Due to these serious influences, many studies have been focused on identifying the causes, reducing the incidence, and extending the effects for several decades.

Some factors were determined to enhance pressure injury, such as lower blood pressure, prolonged immobility, and increased surface pressure due to poor blood circulation to the skin and tissue [4,5,6]. Pressure injury usually occurs in the area of skin and tissue with high pressure, shear, and friction [7]. Studies of the potential causes of pressure injuries have been performed over several decades. The most acceptable model is based on a clear correlation between continuous pressing time and the pressure level reported by Kosiak in 1959 [8]; the model was then improved by Reswick et al. in 1976 [9]. High pressure on the skin surface leads to a less affordable pressing time to prevent pressure injuries; in other words, decreased pressure on the skin surface could lead to a long staying time. The riskiest positions of the human body include the areas of extreme bone prominence, such as the sacral, hip, and heel areas [10]. High interfacial pressure is easily generated due to thinner tissue and corresponding reduced buffer of pressure release. Based on this model, many methodologies, including frequent repositioning every 2 h, static support surfaces, and dynamic support surfaces, were developed to reduce the pressure and prevent pressure injuries. In recent years, interface pressure measurement has become a useful technique to check pressure distribution and reduction [11,12]. A pressure-sensing mattress with a pressure sensor array can be placed between the human body and support surface [12]. After collecting the data from each pressure sensor, a pressure map can be generated by a computer and readout system. However, the efficiency and precision of these developed systems are not fully accepted for real applications because of concerns regarding the cost and reliability of volume pressure sensors. For example, regarding the collection, all interfacial pressure measurements of the human body, a smaller dimension of one sensor, and spacing between neighboring sensors will produce better resolution of the pressure distribution. In this case, the total number and cross talk of pressure sensors will be both increased and follow the large database requirement and low accuracy. To obtain a better operation efficiency for a large database, a readout system with a high efficiency and accuracy is highly demanded.

In general, the working principle of pressure sensors can be classified into three major types: capacitive [13], resistive [14], and piezoelectrical [15]. Among the applications to monitor the interfacial pressure of the human body, the capacitive type pressure sensor on a flexible substrate is the most promising candidate with advantages of high sensitivity and comfortability [13,16]. Due to the natural properties of a flexible substrate, the major interference of these pressure sensors can be the cross-talk from mechanical stress induced by the neighboring area [17]. To reduce external interferences, several methods had been proposed to improve the readout system, such as the adaptive filter [18,19], least squares [20,21], minimum mean square error (MMSE) [22], maximum a posteriori probability (MAP) [23], iterative method [24], Wiener filter [25], and Kalman filter [26]. Regarding the natural properties and application of pressure sensors, the Kalman filter has a superior performance for the readout system with a single variable of input, a linearly response output, a reduction of noise and a high efficiency of prediction [26,27,28]. Therefore, a newly developed readout system integrated with a microprocessor, impedance converter, and algorithm design of the Kalman filter is first proposed and illustrated in the current study for a pressure sensor array of 14 × 18 pixels on a textile-based mattress for clinic interfacial pressure monitoring.

In this study, a systematic study of the readout system improved by a Kalman filter is proposed to minimize the potential impact of noise and cross-talk of a flexible pressure sensor array. With successful optimization, the readout system was applied with a commercial pressure-sensing mattress for a preliminary study between interfacial pressure and pressure injury progress of patients with cardiac operations. This platform can be a powerful smart machine to integrate with active air mattresses for future clinical applications.

## 2. Experimental

**Figure 1:**
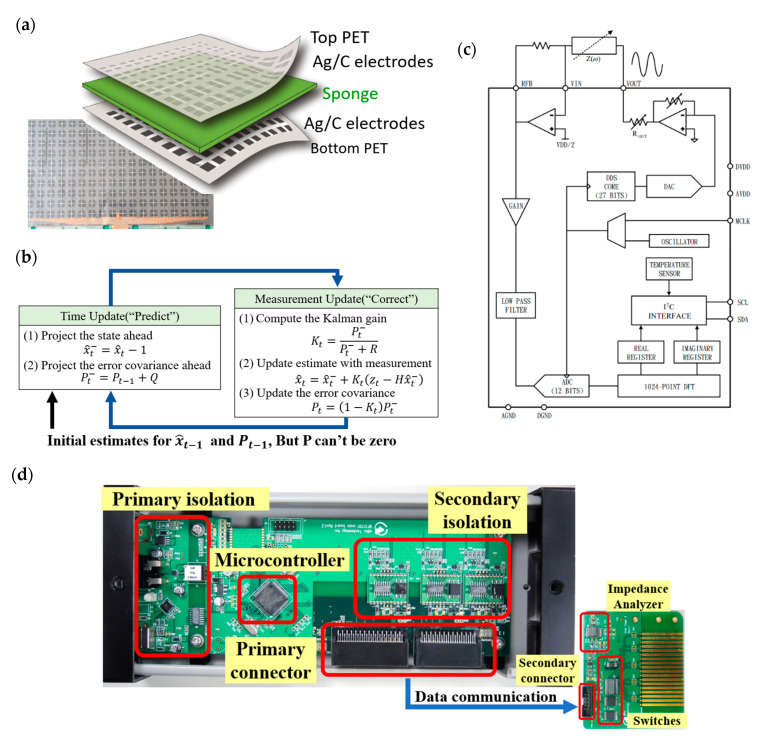
(**a**) Schematic plot and picture of the flexible pressure sensor. (**b**) Operation of the Kalman filter. (**c**) Block diagram. (**d**) Image of the developed impedance readout system.

To measure the interfacial pressure, the pressure sensor array based on a flexible substrate is the basic requirement for human body usage. In the first part, a five-layer capacitive structure with polyethylene terephthalate (PET) for a top and bottom substrate, a two-layer electrode comprising carbon and silver gel screens printed on both PETs, and a sponge (DH60; Chiao Fu Enterprise Co., Ltd., Taichung, Taiwan) for the dielectric layer were assembled by a hot-press process. The signal wires were extended from all electrodes on the PET substrate with defined coordination of the X and Y axes. Combining the selected X and Y coordination of the top and bottom electrode, the interfacial pressure of specific pressure sensors can be calculated from the measured capacitance changes. The dimension of single-pressure sensor is 2.2 × 2.2 cm^2^ with a spacing of 3 cm to the neighboring sensor. The total dimension of this fabricated sensing pad with a dimension of 30.8 × 56.6 cm^2^ for 10 × 18 points was fabricated to investigate basic pressure sensing and the noise interference of capacitive sensors. The picture and schematic plot of this flexible pressure sensor array are shown in Figure 1a. The following section will present the basic operation mechanism of the pressure sensor and impedance measurement system.

The equation of capacitance with parallel electrodes is listed below as Equation (1).

(1) $$C\;=\;\frac{Q}{V}\;=\;\frac{\varepsilon A}{d}$$

where C is the capacitance, *Q* is the sum of charges, *V* is the voltage induced by the accumulated charges, and ε is the dielectric constant of material between 2 parallel electrodes, *A* is the overlap area between 2 parallel electrodes, and d is the distance between 2 parallel electrodes or the thickness of dielectric layer. Once pressure is applied to this parallel-plate capacitance, the distance between 2 parallel electrodes or thickness of the dielectric layer will be decreased to *d*-Δ*d*. The capacitance can be changed using Equation (2).

(2) $$C\;=\;\frac{\varepsilon A}{d-\Delta d}$$

where Δ*d* is the distance change by the applied pressure. The relationship between the applied force and deformation can be expressed by Hooke’s law applied to a flexible pressure sensor, as listed in Equation (3).

(3) $$F\;=\;\;k_{c}\Delta d$$

where *k_c_* is the force constant that can be determined by the material and its geometry, and Δd is the distance of deformation such as the reduced thickness of the flexible capacitance of the pressure sensor. Therefore, Δd equals *F/kc*, which can be applied to Equation (2) to generate Equation (4).

(4) $$C=\frac{\epsilon A}{d-F/k_{c}}$$

In the measurement of the capacitance of the pressure sensor, the impedance, including the resistance and capacitance by the frequency response, is necessary to obtain the actual value. As listed in Equation (5), the relationship between the frequency and deformation of the capacitance of the pressure sensor can be obtained using Equation (4) [29].

(5) $$Z_{c}\;=\;\frac{1}{j2\pi fC}\;=\;\frac{1}{j2\pi f\left(\frac{\varepsilon A}{d-F/k_{c}}\right)}$$

where 2*πf* is the angular frequency, *f* is the frequency and *j* is the expression of $\sqrt{-1}$. Equation (5) can be simplified as Equation (6) with D = 1/*j*2*πfεA* because the values of 2*πf*, ε and *A* are all constants.

(6) $$Z_{c}\;=\;D\left(d-\frac{F}{k_{c}}\right)$$

The higher is the force (F, given pressure), the lower is the impedance (*Z_c_*), which is the basic operation mechanism of the flexible pressure sensor.

To obtain a high efficiency and precision of the flexible pressure sensor, the Kalman filter with the variables of prediction and measurement was applied in the readout system. There are 5 formulas of the Kalman filter from the variance of signal and noise in both prediction and measurement, which are already used for the dynamic system of multi-variables, including electrocardiogram (ECG) [30], electroencephalogram (EEG) [31], robotics and vision [32,33], and industrial applications [27]. Due to the single variable of capacitance, these 5 equations can be simplified as follows:

(7) $$\mathrm{Predicted}\;\mathrm{state}\;\mathrm{estimate}:\;{\hat{x}}_{t}^{-}\;={\hat{x}}_{t-1}$$

(8) $$\mathrm{Predicted}\;\mathrm{error}\;\mathrm{covariance}:\;P_{t}^{-}\;=\;P_{t-1}+Q$$

The previous value of impedance (${\hat{x}}_{t-1}$) is used for the predicted state of impedance $\left({\hat{x}}_{t}^{-}\right)$. The variance of the predicted error ($P_{t}^{-}$) is calculated by the previous value of covariance $\left(P_{t-1}\right)$ added to a factor (*Q*).

(9) $$\mathrm{Kalman}\;\mathrm{gain}:\;K_{t}\;=\;\frac{P_{t}^{-}}{P_{t}^{-}+R}$$

(10) $$\mathrm{Updated}\;\mathrm{state}\;\mathrm{estimate}:\;{\hat{x}}_{t}\;=\;{\hat{x}}_{t}^{-}+\;K_{t}\left(z_{t}-H{\hat{x}}_{t}^{-}\right)$$

(11) $$\mathrm{Updated}\;\mathrm{error}\;\mathrm{covariance}:\;P_{t}\;=\;\left(1-K_{t}\right)P_{t}^{-}$$

where *R* is the variance of current error, *K_t_* is the Kalman gain with the variance predicted error divided by the difference in variance between the current and predicted error, *H* is the corresponding matrix of multi-parameters.$\;{\hat{x}}_{t}$ is the updated impedance in estimation, *z_t_* is the current measured impedance, and *P_t_* is the error variance of the current value. The detailed block diagram of this designed Kalman filter is shown in Figure 1b. Therefore, the value can be closely forecasted and measured using a lower impact of error variance in real applications.

## 3. Results and Discussion

Considering the basic signal of pressure-dependent capacitance, a high-precision impedance measurement system combined with a microprocessor was designed and fabricated. The block diagram of this proposed readout system is shown in Figure 1c. First, the modulated frequency of the input-triggering signal can be generated for the impedance measurement using the direct digital synthesizer. The ac signal with a certain frequency can be used to induce the charges of the measurand capacitor. The impedance and capacitance can be recorded by the integration of charges and then transferred into an analog-to-digital converter (ADC) to calculate digital signal processing. The picture of this developed readout system is shown in Figure 1d. To compare with the impedance results from this developed system, a commercial impedance analyzer, GwINSTEK LCR-817, was also used to measure the capacitance and resistance of flexible pressure sensors.

**Figure 2:**
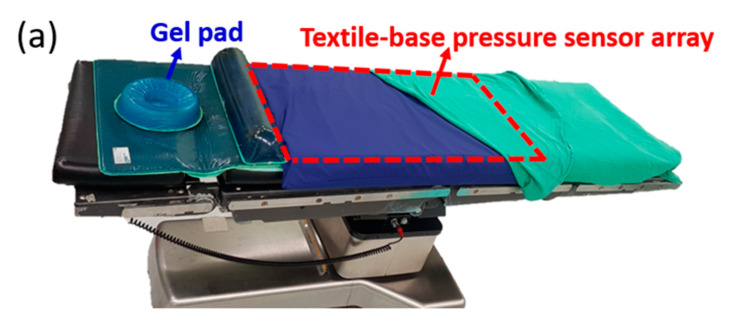

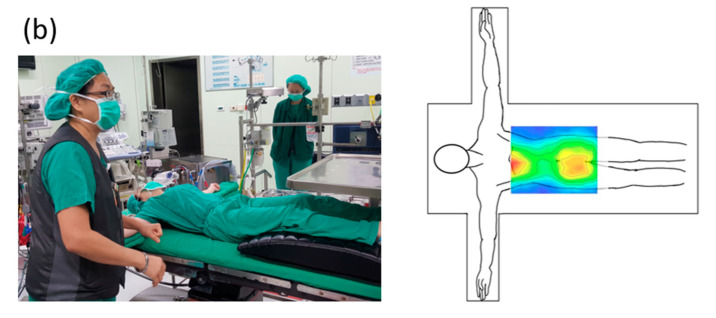
Picture of the real situation for the setup in the operation room: (**a**) A flexible pressure mattress on an operation bed with (**b**) a nurse lying on it.

Based on the concept of this developed PET-based pressure sensor array and readout system, an upgraded commercial product, the textile-based pressure sensing mattress (ePad-ExtraS50, eBio Tech., New Taipei, Taiwan) presented in the second part, was used to replace the PET-based pressure sensor array in the first part for interfacial pressure monitoring embedded on the operation bed. The total dimension of this fabricated sensing pad with a dimension of 67 cm × 138 cm for 14 × 18 points matches the operation bed with a dimension of 50 cm × 200 cm. The area of the whole sensor array is designed 46 cm × 127 cm for the efficiency of data collection and cost saving. The extra area out of sensor array is used to fix on the operation bed. The whole system integrates a pressure-sensing mattress on the operation bed with the data processing unit and a computer under the operation bed. The data of the whole sensing area can be displayed as a color-coded 2-dimensional map, a three-dimensional grid, and a numerical pressure value for each pixel of the pressure sensor. Numerical pressure values are automatically calculated to millimeters of mercury (mm Hg) from a measured digitized number from 0 to 1023 and a calibration curve in the system. Next, pressure mapping can be illustrated for each pixel with the corresponding color gradient in the self-designed software to accelerate the processing time and virtualize for the direct observation by human eyes. The detailed setup of a flexible pressure mattress on the operation bed and testing of a nurse laying on it with a schematic plot of two-dimensional (2D) pressure distribution is shown in Figure 2a,b, respectively. Informed consent was obtained with approval from the Chang Gung Medical Foundation’s Institutional Review Board (IRB) before the test. The IRB number is 201701302B0C501.

**Figure 3:**
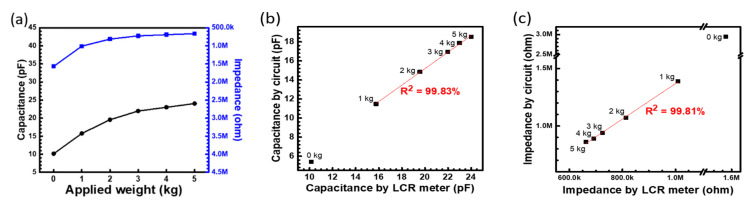
(**a**) Typical response between the applied weight and readout value of the single point of this pressure-sensing pad compared with impedance analyzer. (**b**) Capacitance. (**c**) Impedance correlation between the readout system and impedance analyzer.

As the first part of the readout system evaluation, a standard weight from 0 to 5 kg was applied to the PET-based pressure sensor array to measure the impedance and capacitance using a commercial impedance analyzer and readout system, respectively. The response of the capacitance and impedance are shown in the Y1 and Y2 axis with the different applied weights (Figure 3a). When the applied weight increases, the capacitance increases and impedance decreases because the thickness of the dielectric (the sponge in mattress) decreases. To confirm the measurand values between the circuit-based readout system and commercial impedance analyzer, the values of capacitance and impedance from the 2 systems are plotted, as shown in Figure 3b,c, respectively. The linearity of the correlation curve for 5 points from 1 to 5 kg between these 2 systems is approximately 99.8% for capacitance and impendence, which is acceptable for real application. Both equations show the similar behavior. Therefore these calibration equations can be used to calculate the applied weight by the measured capacitance or impedance. The offset between these 2 systems could be from the embedded resistance and capacitance of the circuit, which can be ignored to have a highly correlative and fast-readout value of pressure changes in the volume pressure sensor array in real applications.

**Figure 4:**
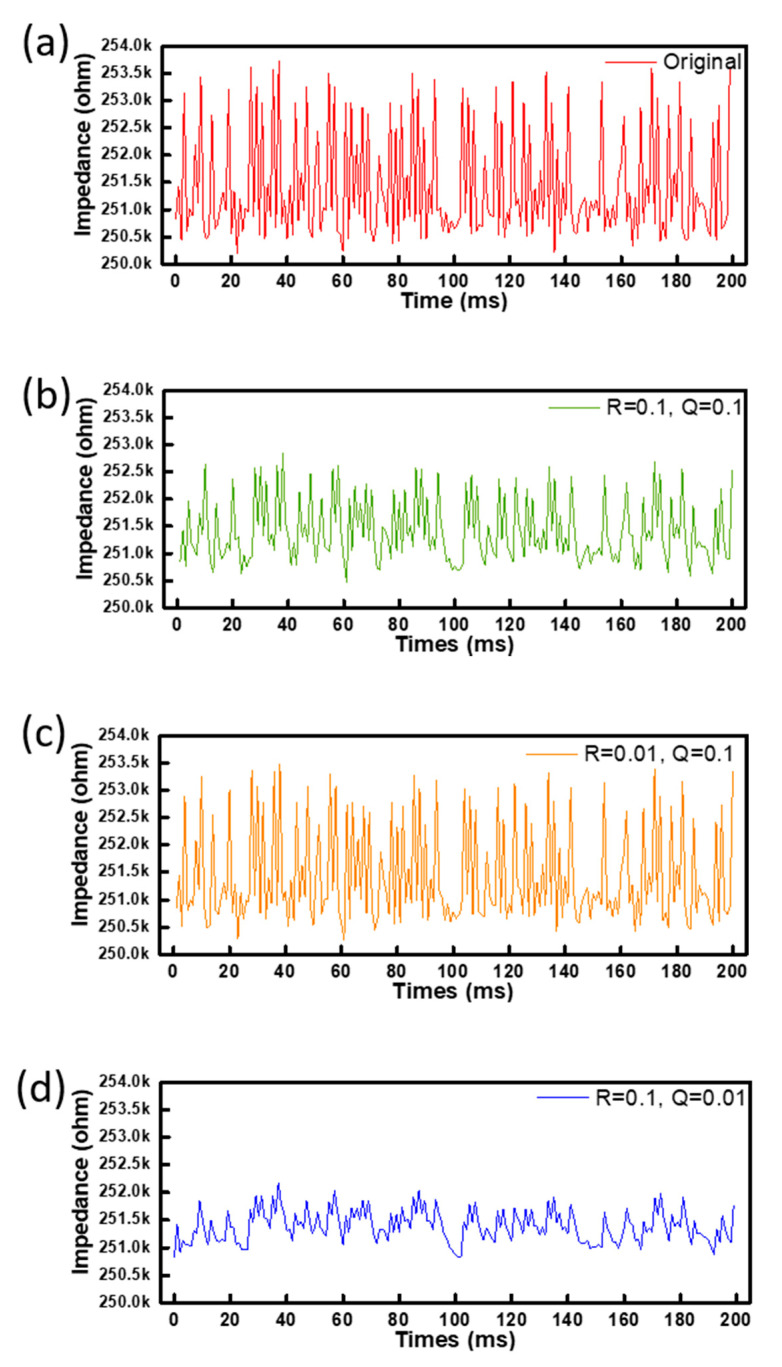
Time-dependent response of the single flexible pressure sensor with (**a**) designed noises of input by the modification of R and Q for (**b**) 0.1/0.1, (**c**) 0.01/0.1 and (**d**) 0.1/0.01 in the Kalman filter.

To verify the function of the Kalman filter, a self-designed random noise with a variance of 0.402% was applied to this designed readout system (Figure 4a). This designed noise is similar to the basic signal variation system level without any pressure loading. Three different groups of the parameter modification for R and Q values were set as 0.1/0.1, 0.01/0.1, and 0.1/0.01, respectively. In the first group of R/Q = 0.1/0.1, the predicted and real variance of error are the same as 0.1. With this setting, the output signal of impedance is shown in Figure 4b with the variance reduced to 0.2284%. Thus, the noise-induced variance can be improved by approximately 43%. In the second group of R/Q = 0.01/0.1, as shown in Figure 4c, the predicted variance of error is 10 times higher than the real variance, making the final variance 0.3565%. The improvement is only 11%, indicating the real variance plays a more important role than the predicted error. In the third group shown in Figure 4d, R/Q = 0.1/0.01 makes a better reduction of variance, that is, 0.1123%. The improvement can be approximately 72%, which can be a good index for the function of the Kalman filter.

**Figure 5:**
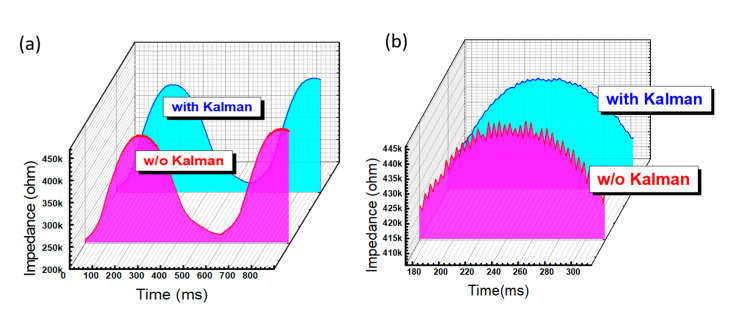
Time-dependent response of the single flexible pressure sensor with a regular pressing force and 24% duty cycle: (**a**) without and (**b**) with the Kalman filter.

To test the improvements of the Kalman filter, a regular pressing force with a step frequency of 1 Hz using a self-designed rotating machine as shown in Figure S1 (Supplementary Materials) was applied to a single-pressure sensor to check the performance. As shown in Figure 5a, the time-dependent response with a sine-wave-like shape can be observed due to the regular movement of this rotating machine inducing the dielectric strain and following the electric response. Low and high impedances can be found when the force has fully left and is then pushed to the sensor surface, as shown in Figure 5a. Some small variations in the periodic vibration can be found as an extra noise added to the major response of the sine wave. This type of noise could be from the sampling rate and data processing speed of the readout system, which may exist in all commercial and portable readout systems. Using this designed Kalman filter with the modifications of R = 0.1 and Q = 0.01, the time-dependent response of the same stepping force can be significantly improved. For a clear comparison, the parts of the high impedance without pressure and with and without the Kalman filter are zoomed in, as shown in Figure 5b. The vibration of the wave form is reduced for the same operation using the Kalman filter. Therefore, this designed Kalman filter was shown to have a better noise immunity and stable readout response of the pressure sensor.

**Figure 6:**
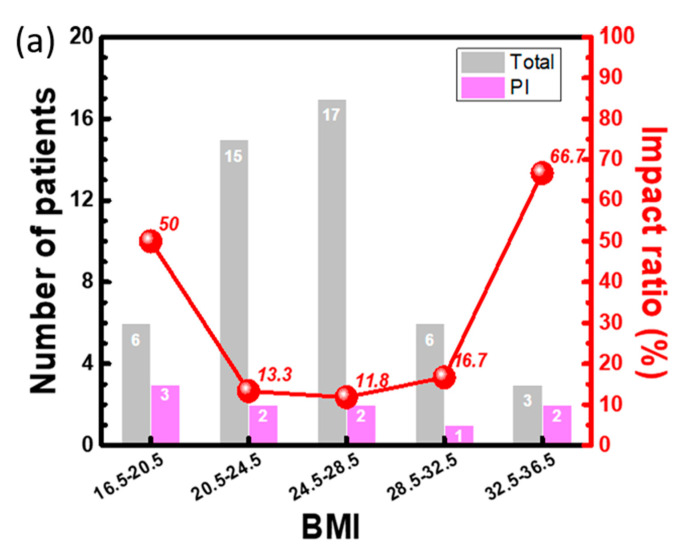

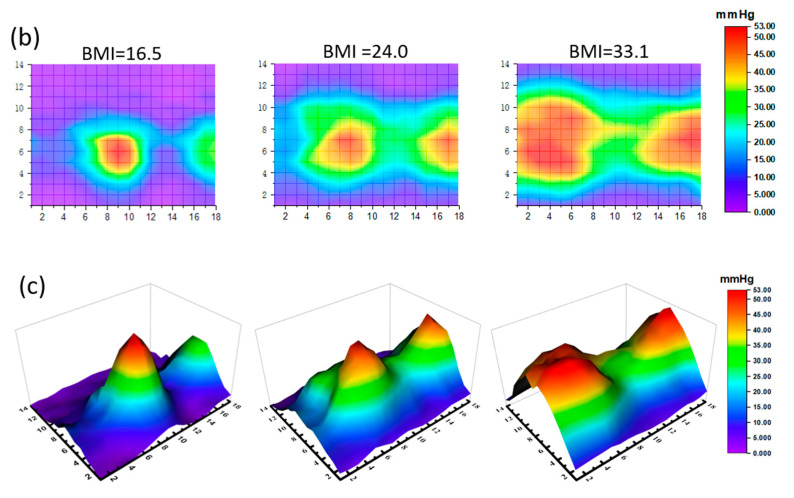
(**a**) Number of patients with and without pressure injury classified by the patient body mass index (BMI). Typical interfacial pressure distribution at the 3rd hour for patients with 3 different BMIs shown in (**b**) 2D and (**c**) 3D images.

To check the potential for clinical application, this proven readout system was used to combine with a commercial flexible pressure mattress (as shown in Figure 2a) to test the long-term interfacial pressure for patients with cardiac operation. The setup of the pressure mattress shown in Figure 2b was prepared for patients who had signed agreements before the cardiac operation mentioned in the IRB. During the cardiac operations, the interfacial pressure data were measured every 5 min and were then recorded in the hard disk of the readout system. All the data were collected for detailed analysis by the visualization of 2D pressure images and time-dependent response of all pressure sensors after the operation. During the experimental period for 18 months, the data of all 47 patients were collected. The detailed information is classified by the status of the pressure injury with their basic body information and BMI as listed in Table 1. Ten of 47 patients had an obvious pressure injury, and the total impact ratio was approximately 21%. A clear difference was observed in the variance of the body weight and BMI for the group with pressure injury. For better comparison, all the patients were divided into 5 groups by BMI from 16.5 to 36.5 with a grouping range of 4 according to the status of pressure injury. The number in each group and ratio to the total patient group with and without pressure injury are listed in Table 2. The extremely low and high BMI groups have more patients with pressure injury than the normal BMI group; a normal distribution of patient number versus the BMI group was observed for non-pressure injury patients. For better comparison, the total patients and patients with and without pressure injury in each group were assessed (Figure 6a). The distribution of the total number of each group marked in gray color was similar to a normal distribution by BMI group. The number of patients with a pressure injury from 1 to 3 was comparable in all groups. To further evaluate the impact ratio of each group, the distribution curve of each group was drawn, as shown in the Y2 axis of Figure 6a. The impact ratios of pressure injury in the lowest and highest BMI groups were 50% and 67%, respectively. These 2 ratios were considerably higher than those of other 3 groups with impact ratios lower than 20%, representing a new finding for high-risk patients. To further study the distribution of the interfacial pressure between the BMI groups, typical 2D mapping of the relative pressure of the typical response measured from 3 patients with BMIs of 16.5, 24.0, and 33.1 was performed (Figure 6b). The purple and red colors indicate the low and high interfacial pressure from 0 to 53 mmHg, respectively. High interfacial pressure in red color can be found in the sacral and back areas. Three-dimensional (3D) images of the same data were generated (Figure 6c). The normal case for patients with a BMI of 24.0 can be used to present the standard distribution of interfacial pressure. In the low-BMI patients, the total purple and blue areas were considerably larger than those of the other 2 groups, a finding that can be explained by the low body weight. The difference between the low and high interfacial pressure areas is relatively high (e.g., the slope of the interfacial pressure to distance), which can be explained by the low body weight widely distributed but highly concentrated in the sacral area due to less protection by a thin skin. This natural behavior of patients with a low BMI may increase the risk of pressure injury, which can be treated with special clinical care to prevent future pressure injury. In patients with a BMI = 33.1, the total interfacial pressure measurements were higher than those in the other 2 groups, resulting from the heavy body weight. With this higher interfacial pressure, the risk of pressure injury is also high, corroborating the findings in previous literature [34]. The highest interfacial pressure occurred in the sacral area for all 3 patients with a similar value of ~50 mmHg, similar to the natural behaviors observed in the clinic and literature.

**Figure 7:**
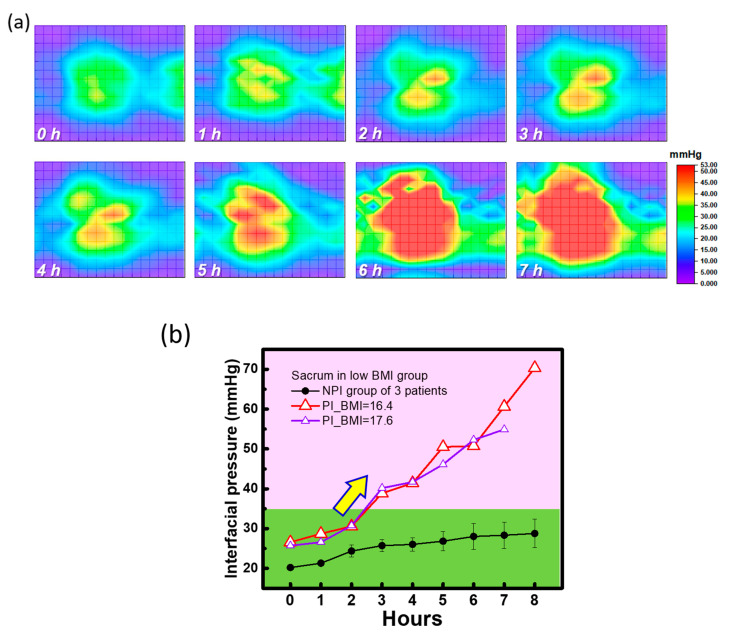
(**a**) Time-dependent 2D pressure image for a typical patient with a low BMI and pressure injury and (**b**) time-dependent pressure trending of the sacrum area for patients with and without pressure injury according to the real measurement in cardiac operations.

Due to the long-term monitoring and recording of interfacial pressure by this readout system, 2D images of the interfacial pressure mapping of a patient with pressure injury can be generated using the time domain to investigate the different risk levels for patients in the same BMI group. As shown in Figure 7a, the time-dependent 2D interfacial pressure mapping of a low-BMI patient with pressure injury demonstrated by a period of 1 h can be used to quickly assess the changes during cardiac operation. The interfacial pressure is slightly increased before 2 h (e.g., less red area) and dramatically increased after 2 h (more red area). Additionally, the impacted high interfacial area shown in red is extended from the sacral to back areas. These areas with high interfacial pressure are riskier than other areas due to the weight distribution by the interaction between bone and skin. Similar measurements of the interfacial pressure of a patient with low BMI and without pressure injury were performed, and all the 2D interfacial pressure images are shown in Figure S2 (Supplementary Materials). The increase in interfacial pressure was relatively low. To fairly compare the time-dependent interfacial pressure between patients with and without pressure injury, the interfacial pressures of the sacral area of 2 patients with pressure injury (called the PI group) and 3 patients without pressure injury (called the NPI group) with the extreme low BMI were measured as shown in Figure 7b. The data of one patient in PI group is firstly screened from original grouping with 3 patients due to the extreme old age (>71 years old) and short operation time (<5 h). Then both patients with pressure injury had a high interfacial pressure level that dramatically increased after 2 h in operation compared with the NPI group. Although the BMI levels of these 2 patients and the NPI group were similar, the interfacial pressure generated by the critical area, such as the sacrum, plays a dominant role in pressure injury. Therefore, the risk of pressure injury is relative to the high interfacial pressure determined by the personal body properties and then increases with the operation time, especially with no pressure redistribution for a long time. Even with a standard pressure release procedure using gel pads inserted between the patient body and mattress, the impact ratio on pressure injury was still 21% in patients with the long-term cardiac operation. This proposed methodology, including the developed interfacial pressure sensing system, and combined with a flexible textile-based pressure sensor array, can be used to classify high-risk patients for pressure injury before long-term operation. Furthermore, pressure redistribution using the tunable air mattress integrated with this proposed flexible textile-based pressure sensing system can be suggested to minimize the interfacial pressure during operation, reducing the impact of pressure injury. Additionally, operation of the tunable air mattress should be carefully considered and evaluated based on the clinical demands of operation, especially for patient safety.

## 4. Discussion

A readout system integrated with the concept of the Kalman filter and impedance measurement is designed and fabricated for a pressure sensor array. The noise immunity can show a 72% reduction by a proper setting in the R and Q factors of this designed Kalman filter. Additionally, the readout signal can have a low variation in a continuous monitor with regular stepping of 1 Hz using this designed Kalman filter. This improvement makes the measurement of the capacitive-type pressure sensor more efficient and accurate, especially for large sensor arrays in real applications. Furthermore, this readout system integrated with a special designed textile pressure-sensing mattress with 14 × 18 pixels for the operation bed was first applied for patients with cardiac operations to collect the long-term interfacial pressure including the 2D mapping and time-dependent response. As listed in Table 3, several pressure sensing mattresses with its readout system are compared. This developed system owns a reasonable sensor pitch and sensor number which makes less crosstalk, interference and system loading. In the meantime, the digitized level of output signals is improved to 10 bits as 1024 levels, which makes the possibility of high-resolution observation. With engineering improvements, the continuous interfacial pressures of 47 patients in the cardio operations were collected for clinic investigations is firstly presented in the same work due to a close cooperation between experts in various fields and this proposed integrated platform. Compared with the basic information of patients and their interfacial pressure behaviors, a high impact ratio of pressure injury can be found in the patients with extreme BMI (e.g., BMI > 32.5 or < 20.5). A clear increase in the slope of interfacial pressure to time can be found after 2 h, even with the protection of a standard gel pad setup in the cardiac operation. Considering these clear results, this readout system is beneficial for early warning, long-term monitoring and large-scale pressure sensor array measurement, which can be applied in clinical settings. Thus, the extra pressure redistribution methodology for high-risk patients, such as the present specially designed air mattress, can be implemented in long-term operations or long-term care settings to reduce the impact of pressure injury in the future.

## Figures and Tables

**Table 1:** Basic information of patients with and without pressure injury.

	n	Male	Female	Height	Weight	BMI	Age	Operation Time (hr)
PI *	11	6	4	163.4 ± 12.9	66.2 ± 18.5	24.7 ± 6.1	54.4 ± 8.1	7.9 ± 2.2
NPI **	37	26	11	163.3 ± 10.3	66.8 ± 11.8	24.9 ± 3.5	58.1 ± 12.1	8.0 ± 2.4
Total	47	32	15	163.3 ± 13.2	66.7 ± 13.2	24.9 ± 4.1	57.3 ± 11.4	8.0 ± 2.3

* PI: Pressure Injury; ** NPI: Non-Pressure Injury.

**Table 2:** Patient number with and without pressure injury classified by 5 different body mass index (BMI) groups.

Ratio (%) & [Total Patient Number]					
BMI	16.5–20.5	20.5–24.5	24.5–28.5	28.5–32.5	32.5–36.5
PI *	30 [3]	20 [2]	20 [2]	10 [1]	20 [2]
NPI **	8.1 [3]	35.1 [13]	40.5 [15]	13.5 [5]	2.7 [1]

* PI: Pressure Injury; ** NPI: Non-Pressure Injury.

**Table 3:** Comparison of pressure sensing systems.

Type Name/ Company	Sensor Specification			System Specification		Clinic Test	Year/ Ref.
	Substrate Type	Sensor Pitch (cm)	Total Sensor no.	Digitized Level	Accuracy (%)	Patient no.	
*CONFORMat@/* *NITTA corp.*	Flexible	2.17	32 × 32	N/A	±10%	0	2011/[35]
*5315/Tekscan Inc.*	Flexible	1	42 × 48	256	±10%	0	2015/[12]
*PX100/* *XSENSOR Tech.*	Flexible	1.27	64 × 160	N/A	±10%	0	2015/[11]
*Mark III/* *Talley Group Ltd.*	N/A	3	12 × 8	N/A	N/A	0	2017/[36]
*ePad-ExtraS50* */eBio Tech.*	Textile	3.2	14 × 18	1024	±8% [±0.2%] *	47	This work

* Accuracy of system without pressure loading can be ±0.2% by the help of Kalman filter.

## Supplementary Materials

The following are available online at https://www.mdpi.com/1424-8220/20/16/4619/s1, Figure S1: Picture of a regular pressing force by the mechanical setup for the single-pressure sensor, Figure S2 Time-dependent 2D pressure image for a typical patient with low BMI and without pressure injury.
