# Supplementary material for: Flexible Textile-Based Pressure Sensing System Applied in the Operating Room for Pressure Injury Monitoring of Cardiac Operation Patients
Source: Sensors (Basel). 2020 Aug 17;20(16):4619. doi: 10.3390/s20164619 (PMC7472060; doi:10.3390/s20164619)
Supplement: Supplementary file 1 [file sensors-20-04619-s001.pdf]

sensors

Article

Flexible Textile-Based Pressure Sensing System Applied in the Operating Room for Pressure Injury Monitoring of Cardiac Operation Patients

De-Fen Shih [1,†], Jyh-Liang Wang [1,2,†], Sou-Chih Chao [1,2], Yin-Fa Chen [3], Kuo-Sheng Liu [4], Yi-Shan Chiang [5], Chi Wang [5,6], Min-Yu Chang [5,7], Shu-Ling Yeh [5,8], Pao-Hsien Chu [9], Chao-Sung Lai [10,11,12,13], Der-Chi Shye [1,2], Lun-Hui Ho [5,8,*] and Chia-Ming Yang [3,10,11,14]

[1] eBio Technology Inc., Xinzhuang, New Taipei City 242, Taiwan; stephanie@ebio-health.com (D.-F.S.); joewang@mail.mcut.edu.tw (J.-L.W.); willson@ebio-health.com (S.-C.C.); DCS19680323@gmail.com (D.-C.S.)
[2] Department of Electronic Engineering, Ming Chi University of Technology, New Taipei 243, Taiwan
[3] Institute of Electro-Optical Engineering, Chang Gung University, Taoyuan 333, Taiwan; chenbearfa@gmail.com (Y.-F.C.); cmyang@mail.cgu.edu.tw (C.-M.Y.)
[4] Department of Cardiac Surgery, Chang Gung Memorial Hospital, Linkou 333, Taiwan; liuks@me.com
[5] Department of Nursing, Linkou Chang Gung Memorial Hospital, Linkou 333, Taiwan; robelae@cgmh.org.tw (Y.-S.C.); gigy@cgmh.org.tw (C.W.); yu@cgmh.org.tw (M.-Y.C.); q22122@cgmh.org.tw (S.-L.Y.)
[6] Department of Nursing, Chang Gung University, Taoyuan 333, Taiwan
[7] Department of Nursing, Oriental Institute of Technology, New Taipei City 220, Taiwan
[8] Department of Nursing, Chang Gung University of Science and Technology, Taoyuan 333, Taiwan
[9] Department of Cardiology, Chang Gung Memorial Hospital, School of Medicine, Chang Gung University, 199 Tung Hwa North Road, Taipei 105, Taiwan; pchu@cgmh.org.tw
[10] Department of Electronic Engineering, Chang-Gung University, Taoyuan 333, Taiwan; cslai@mail.cgu.edu.tw
[11] Biosensor Group, Biomedical Engineering Research Center, Chang Gung University, Taoyuan 333, Taiwan
[12] Department of Nephrology, Chang Gung Memorial Hospital, Linkou 333, Taiwan
[13] Department of Materials Engineering, Ming-Chi University of Technology, New Taipei City 243, Taiwan
[14] Department of General Surgery, Chang Gung Memorial Hospital, Linkou 333, Taiwan
* Correspondence: ho1180@cgmh.org.tw; Tel: +886-3-3281200 (ext. 2811)
† D.-F. Shih and J.-L. Wang contributed equally to this work.

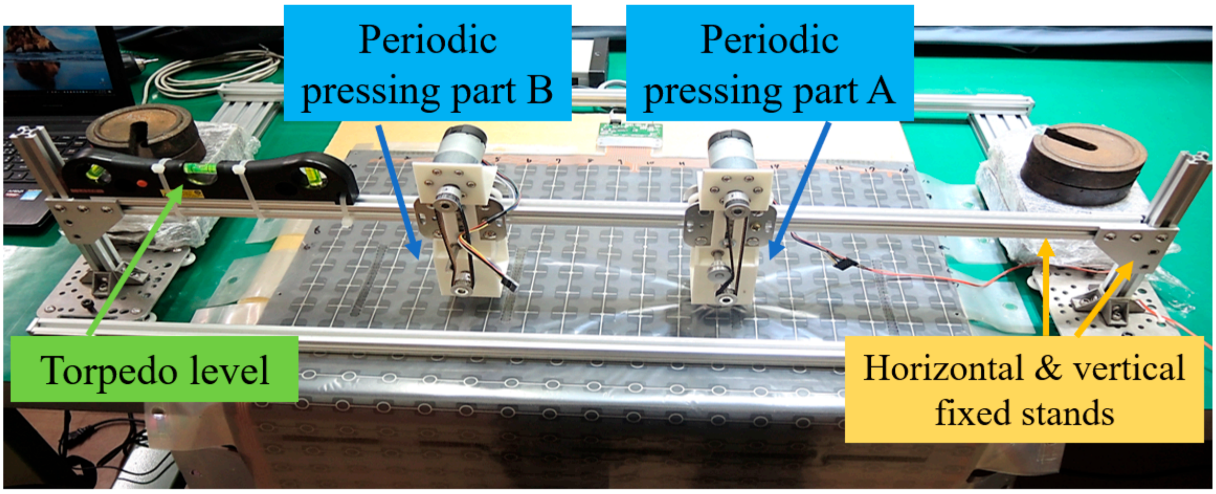

Figure 1. Picture of a regular pressing force by the mechanical setup for the single-pressure sensor.

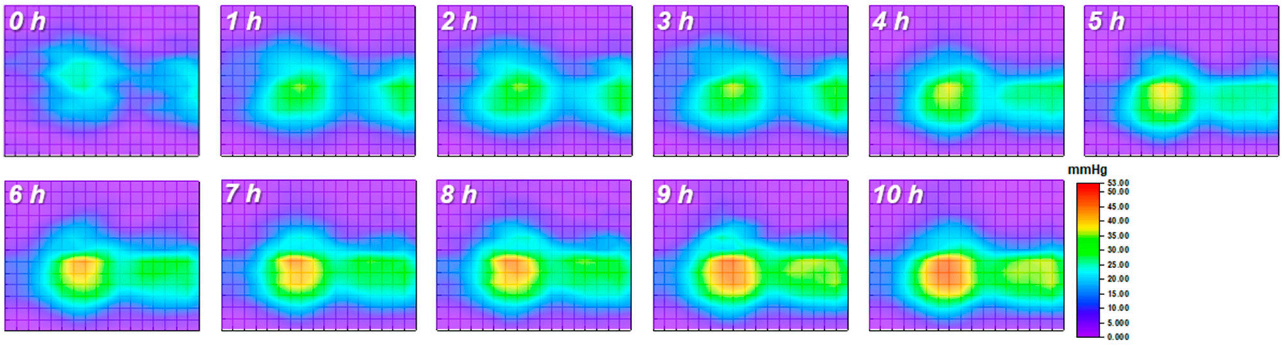

Figure 2. Time-dependent 2D pressure image for a typical patient with low BMI and without pressure injury.

© 2020 by the authors. Licensee MDPI, Basel, Switzerland. This article is an open access article distributed under the terms and conditions of the Creative Commons Attribution (CC BY) license (http://creativecommons.org/licenses/by/4.0/).
